# Neuroanatomical data collection and usability

**DOI:** 10.3389/fnana.2023.1183883

**Published:** 2023-05-19

**Authors:** Javier DeFelipe

**Affiliations:** ^1^Laboratorio Cajal de Circuitos Corticales, Centro de Tecnología Biomédica, Universidad Politécnica de Madrid, Madrid, Spain; ^2^CIBERNED, Centro de Investigación Biomédica en Red de Enfermedades Neurodegenerativas, Madrid, Spain; ^3^Instituto Cajal, Consejo Superior de Investigaciones Científicas, Madrid, Spain

**Keywords:** quality of data, sharing of data, open access data, collaboration between laboratories, interdisciplinary collaboration

We are drowning in a sea of data and starving for knowledge. The biological sciences have exploded, largely through our unprecedented power to accumulate descriptive facts... We need to turn data into knowledge and we need a framework to do it.

—Taken from the 2002 Nobel lecture of Sydney Brenner “Nature's Gift to Science”

In recent years, open access to share data production is commonly becoming required (or encouraged) to prepare a grant proposal or to publish research articles, following FAIR principles (findable, accessible, interoperable, and reusable). Open access to data is without question a highly recommended, sensible way to strengthen science as it allows you to use data generated by others, which may be very expensive and difficult to obtain, providing data that is reusable for your own research goals, and avoiding repetition of experiments. Furthermore, in some cases, it may be necessary to have access to the original data to reproduce published studies that could have a considerable social impact, such as the discovery of a drug that cures a certain disease or when an unexpected discovery is made that represents a radical change in the field.

Setting aside these important aspects of data production and management, the words of Sydney Brenner that appear in the header of this article remind us that science does not simply involve *obtaining* data, but rather transforming it into knowledge. This transformation is one of the major difficulties we must face when studying the brain. Understanding the brain requires data of multiple types to be obtained, including molecular, genetic, anatomical, and physiological. We also need informatics tools and computational neuroscience to integrate different datasets and to account for the data, make predictions and look for new hypotheses to discover new aspects of the structural and functional organization of the brain. These assertions seem clear for everyone studying the nervous system. However, there are a number of additional critical difficulties that we must tackle in all fields of research regarding data production and usability. What follows is my personal point of view on this subject from a neuroanatomical perspective, although it could probably be applied to any discipline.

The first issue is the quality of available neuroanatomical data. How good or bad the data is depends on several factors. One of the most important is the great variation in the quality of the light and electron microscopic preparations produced in different laboratories using any of the large variety of techniques available at present. The variations in quality depend on the fixation, preparation and processing of brain tissue, as well as on the techniques used to visualize neurons, glia, synapses, etc. Another factor is the high interindividual variability that exists regarding a number of the structural and functional attributes of the brain in general and the human brain in particular. This variability includes inter-subject variability with respect to brain size, shape and cytoarchitecture. In a given brain region, there are also variations in the structure of individual neurons and glia as well as in the number and density of synapses, types of synaptic targets, etc. It has been proposed that some of these variations might be explained by differences in age and sex, among other features. However, these sources of variability are frequently not included in the experimental design and/or in the interpretation of the data. These factors might explain why, for example, the density of neurons, glia, and synapses in a given brain region often varies significantly between different studies. In other words, the reliability of the data varies. Thus, we need to have a deep understanding of the techniques and experimental design used to obtain the data.

The second issue is the large volume of data provided by the enormous number of published articles over the years and by the increasing rate of articles that are produced each year. It is important to note that these articles largely remain unread in their original form. Considering both issues together, that is, quality of data and superficial analysis of published data, a consequence is that it is relatively common to accept or reach conclusions that may be erroneous but take root in the scientific community, giving rise to “noise” that blurs our knowledge of the brain.

These problems must be taken seriously but dealing with them is challenging. In many cases, detailed reading of the scientific literature is not enough since the raw data is not available, or there is a lack of information about important methodological details, or the user lacks the expertise required to evaluate the significance and quality of data. One solution is to promote collaboration between laboratories to truly achieve full access to data and transparency of methodological issues. Since in general the research focus, expertise and methodological approaches differ greatly between laboratories, the efficacy of this collaboration will depend not only on the understanding of how data is collected and on how robust it is, but also on substantial communication between all the research teams. In order to try to put this into practice, the EU-funded Human Brain Project (HBP) created a digital neuroscience research infrastructure, named EBRAINS (https://ebrains.eu/), which could be particularly useful to continue to promote collaboration between laboratories.

In conclusion, to advance our knowledge of the brain more quickly and efficiently, we need to improve the usability of existing data and the production of new data. The best strategy is to harness the added value provided by multiple laboratories working together—each with different areas of expertise but pursuing the same specific goal. In this way, ambitious objectives addressing a well-defined scientific question can be achieved. Therefore, it is imperative to promote interdisciplinary collaboration and data sharing, at both national and international levels, to make better use of the expertise and resources of individual laboratories.

## Author contributions

The author confirms being the sole contributor of this work and has approved it for publication.

